# SAW: A Method to Identify Splicing Events from RNA-Seq Data Based on Splicing Fingerprints

**DOI:** 10.1371/journal.pone.0012047

**Published:** 2010-08-10

**Authors:** Kang Ning, Damian Fermin

**Affiliations:** Department of Pathology, University of Michigan, Ann Arbor, Michigan, United States of America; Centre de Regulació Genòmica, Spain

## Abstract

Splicing event identification is one of the most important issues in the comprehensive analysis of transcription profile. Recent development of next-generation sequencing technology has generated an extensive profile of alternative splicing. However, while many of these splicing events are between exons that are relatively close on genome sequences, reads generated by RNA-Seq are not limited to alternative splicing between close exons but occur in virtually all splicing events. In this work, a novel method, SAW, was proposed for the identification of all splicing events based on short reads from RNA-Seq. It was observed that short reads not in known gene models are actually absent words from known gene sequences. An efficient method to filter and cluster these short reads by fingerprint fragments of splicing events without aligning short reads to genome sequences was developed. Additionally, the possible splicing sites were also determined without alignment against genome sequences. A consensus sequence was then generated for each short read cluster, which was then aligned to the genome sequences. Results demonstrated that this method could identify more than 90% of the known splicing events with a very low false discovery rate, as well as accurately identify, a number of novel splicing events between distant exons.

## Introduction 

Alternative splicing is a process by which exons of genes are differentially recombined at the messenger RNA level to produce various transcripts, enabling the a single gene to encode for multiple protein products. There are numerous modes of alternative splicing, and they have greatly increased the range of proteins that can be encoded [1]. It is predicted that in humans, over 80% of genes are alternatively spliced [2]. Recent work has shown that splicing events are much more prevalent than previously thought [3], [4], [5]. Splicing event identification is crucial for transcription analysis [6]. Traditionally, identifications were made by aligning gene sequences against EST sequences [6], [7], [8] or by microarray [2], [6], [9]. Recently, RNA-Seq has emerged as the next revolution in sequencing technology enabling comprehensive transcription profiling with unprecedented precision and a low error rate [3], [5], [10], [11]. Leveraging this technology will allow for a far more comprehensive analysis of splicing events.

The most commonly used method for identifying novel splicing events using RNA-Seq is to compile a junction library. This library is constructed based on either exon models and contains all known and predicted splice junctions. RNA-Seq reads are then mapped to the library [3]. Short reads from RNA-Seq that map to sequences in a junction library are evidence for a splice junction. These libraries are of limited use, however, since they constructed from exons that are relatively close to each other on the genome. Such libraries can only be used to identify alternative splicing within genes or splicing events between exons which are in close proximity [10] [12].

Identifying novel splicing events involving distant exons is very challenging [3]. Constructing junction libraries to encompass all potential splicing events are impractical since the search space explodes with the increasing number of potential splicing partners. Since the splice site is not known in advance, the laborious spliced alignments of short sequence reads against gene sequences further complicates the process and makes splicing event identification highly error prone.

In this work, we propose a novel method we call splicing even identification by absent words (SAW). This approach has the potential to efficiently identify novel splice junctions including those involving distant exons. Our approach begins by extracting unique sequences that define a splice junction (termed splicing fingerprints), and clusters short reads to these fingerprints. Short reads for splicing events are then clustered based on the minimal absents words [13] from gene sequences that we demonstrate to be fingerprints of these splicing events, which. Thus we have proposed a novel method, SAW, which is able to cluster short reads and identify candidate splicing sites based on minimal absents words before the alignment of short reads to genome sequences. Since both short read clusters and candidate splicing sites were predicted before alignment, there was no need to construct the junction library. As discussed earlier, not having to assemble an unwieldy junction library that must capture known and predicted junction sequences is an enormous step forward in productivity. The SAW method improves efficiency to an extent that addresses the huge obstacles previously mentioned and makes the comprehensive identification of splicing events between two distant exons possible.

## Methods

Given the complex jargon associated with this work a brief describing summary is presented here.

Splicing event identification by absent words (SAW) is distinguished from traditional splicing event identification methods based on searching junction libraries. The first stage in the SAW pipeline is to collect short unique sequences (termed fingerprints) that represent all possible splice events between exons. After this acquisition, short reads are mapped to the fingerprints and clustered. From these clusters a consensus sequence is derived that maximally covers the fingerprint sequence. Candidate splice junctions can then be identified from these clusters. The final step is to align the consensus sequences against the reference genome for the identification of splicing events.

### Definitions and Theorems

This section defines absent words and minimal absent words. Following this, a theoretical analysis demonstrates that fingerprints that might represent splicing events are minimal absent words (MAWs) from the reference sequences. In the context of this work, our reference sequence is the genome of the organism of interest.

**Absent words:** Broadly speaking, absent words are strings that are not present in a given body of text. In the context of biology, our text could be any type of biological sequence data. Absent words from gene models have been a recent focus of research since these absent words may correspond to lethal mutations [13]. In this study, we looked at absent words from a different angle: absent words that cannot be mapped to the genome may correspond to sequences only arrived at through splicing events.

A subclass of absent words are minimal absent words (MAWs) [13]. These are absent words for which the removal of a single letter from either end of the word produces a new word that can be aligned to the reference genome. As an example consider this sequence *S* (Example 1):
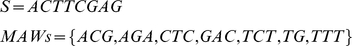
In the above example, *S* represents our sequence, and the set called *MAWs* represents all of the MAWs for *S*. None of the words in MAWs are substrings of the genome *S*. However, by dropping a single letter from any of end of the words in *MAWs* you arrive at a new set of words all of which can be aligned to *S*. For example, dropping the first or last letter from word *GAC* would result in *GA* and *AC*, both of which are substrings of *S*.

**Lemma 1**: An absent word is itself an MAW or a superstring of at least one other MAW.

**Proof**: Suppose the absent word is a*S*b, where *S* is a string of some arbitrary length, and a and b are single characters from the same alphabet. Assume further that a*S*b is not an MAW and not a superstring of any MAW. Then according to the definition of MAW, either a*S* or *S*b is also an absent word. If a*S* (or *S*b) is an absent word, then there is no substring of a*S* (or *S*b) that is a MAW. This fragmentation can continue until a*S*b = x*C*y, for which x and y represent nil or substring of a*S*b, and *C* is a single character. According to the assumption, C represents absent words but is not a MAW. However, this cannot be true given previous logic. Therefore, the assumption that a*S*b is not a superstring of any MAW is not true: either a*S*b is a MAW itself, or there is at least one MAW which is the substring of absent word a*S*b.

Based on Lemma 1, it is possible to see that every absent word corresponds to at least one MAW as itself or its substring. It is also probable that an absent word is a superstring of multiple MAWs.

**Lemma 2**: If the reverse compliment is considered for finding MAWs, then the reverse complement of a MAW is also a MAW.

**Proof**: Consider the same construct of a*S*b described above. Its reverse compliment is b′*S′*a′, where a′, b′ are the complimentary base pairs of a and b, respectively, and *S′* is the corresponding reverse compliment of *S*. If a*S*b is an MAW, then both a*S* and *S*b are present in reference sequences, therefore, b′*S*′ and *S*′a′ are also present in reference sequences in a reverse compliment form. Therefore, b′*S′*a′ could either be present in reference sequences, or it is a MAW. If b′*S′*a′ is also present in forward or reverse compliment form, then a*S*b is also present, which contradict to the assumption that a*S*b is a MAW. Therefore, b′*S′*a′ is also a MAW.

Take the previous example (Example 1), when reverse compliment is considered for finding MAWs, then
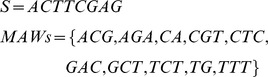
In which the reverse compliments of MAWs “ACG” and “CAG” are also MAWs for sequence S.

Lemma 2 shows the dual existence property of MAW: when reverse compliment is considered, which is a common scenario in analyzing genomic sequences, either a word and its reverse compliment are both MAWs or they are simultaneously not present in the reference sequences.

MAWs that are not present in gene models may correspond to sequences for splice events that are unknown. Therefore, they are the possible finger prints for novel splicing events.

**Short reads for alternative splicing:** Using next generation sequencing techniques, a comprehensive profile of alternative splicing events has been compiled based on short reads. Most of the short reads align within one complete exon. A smaller set of the short reads map to known exon-exon junctions. However, there are many short reads that are not aligned to any known gene models. These short reads might well be annotation errors, but there are still many of them which may correspond to (a) novel exons, (b) novel splicing events or (c) sequences that represent mutations in genes that are not present in known gene models.

Our focus here is on those short reads that cannot be mapped to the genome. These may correspond to novel splicing events between distant exons. It is easy to see that short reads that do not map perfectly (i.e.: without mismatches) to any known regions of the genome are by definition absent words in the context of the genome sequence. According to Lemma 1 each of these short reads corresponded to at least one MAW from a gene model.

**Theorem 1 (Fingerprint theorem)**: Consider a short read with the following properties:

- It does not align to any part of the genome
- It does align to a splice junction site
- It contains at least one MAW within its sequence

Given these properties of a short read, at least one of its MAWs must straddle the splice junction.

Here we give an example (Example 2) to illustrate Theorem 1. Suppose there are only two exons in the reference genome of interest. The reference genome G, exon sequences E_1_ and E_2_, and the MAWs for G are
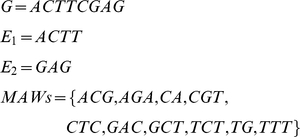
Suppose the short reads are of length 4, then all of the possible unmapped short reads that correspond to the splicing event between E_1_ and E_2_ are in the set of {CTTG, TTGA, TGAG, CAAG, TCAA, CTCA} in both forward and reverse compliment form. All of these short reads contain the MAW “CA” or “TG”, both of which straddle the splicing junction.

Theorem 1 indicates that for identifying splicing events using short reads, the MAWs play an important role in identifying the splicing event. On the basis of Theorem 1 a large number of short reads that do not have any MAW as their substrings can be filtered out even without trying to align them to genome sequences.

The sites between any of the two characters in a sequence are referred to as “boundary sites”. For example, two boundary sites for the sequence “GAC” are between G|AC and between GA|C. In this work it is assumed that splicing events can only occur at the boundary sites of the exons, as is generally case for most of splicing events.

**Lemma 3**: If a short read contains only one MAW, and that short read straddles a single splice junction, then the splice junction must be fully contained within the boundaries of the MAW.

**Proof**: Consider a short read that straddles a single splice junction and encompasses a single MAW. If the junction site lies outside of the MAW's boundaries, then the MAW must be fully contained within one of the two splice site exons.

In Example 2 above, the only splicing site between exon E_1_ and E_2_ is between T-G (or C-A in reverse compliment form), which are inside of the “boundary sites” of MAW “TG” and “CA”.

Based on Lemma 3, all possible candidate splice sites identified by short reads, should be straddled by at least one MAW.

**Lemma 4**: If a short read contains two MAWs that are not overlapping, then this short read can not correspond to just one single splicing event.

Lemma 4 is derived from Lemma 3. If a short read contains two MAWs that are not overlapping, then either this short read does not correspond to any splicing event, or it correspond to two splicing events. However, if two MAWs overlap on a short read, then this short read may still correspond to a single splicing event.

**Lemma 5**: If a short read corresponding to a splicing event contains multiple overlapping MAWs, then the splice site must be located within the consensus portion of the overlapping MAWs.

Lemma 5 is derived from Lemma 3 and Lemma 4.

**Theorem 2 (Splice site theorem)**: Consider a short read with the following properties:

It does not align to any part of the genome
It does align to a splice junction site
It contains at least one MAW within its sequence

the corresponding splice junction can only reside on a MAW for this short read that does not overlap with other MAWs, or the overlapping part of the MAWs for this short read.

This Theorem is derived from lemma 4 and 5. For example, suppose the short read is ACCGGCACT, and the MAWs are {ACC, GCA, CAC}, then there are only 3 possible splicing site, which aer indicated at vertical bars in “*A|C|C*G*GC|AC*T”.

**Figure 1:**
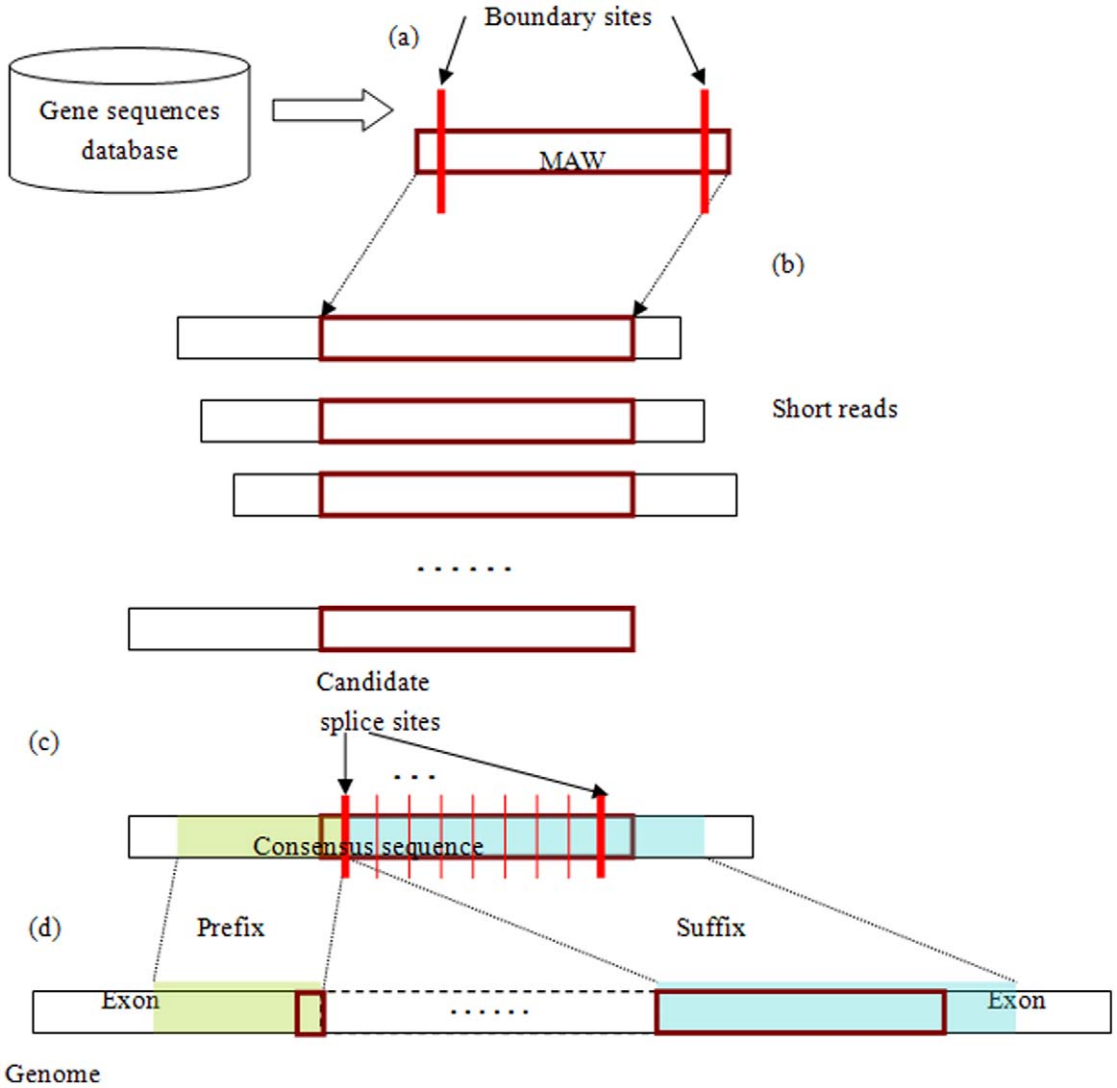
Clustering short reads and determination of candidate splice site by MAWs. (a) Extraction of MAWs from gene sequences database. (b) Mapping MAW onto short read. (c) Clustering of short reads based on MAW to form consensus sequences. (d) Aligning consensus sequence to exon boundaries according to candidate splice site. Only consensus sequence (in color) is used to align against exon boundaries. Annotation: shadowed area indicates consensus sequence.

Theorem 2 explicitly gives all possible splicing sites on MAWs, if this MAW corresponds to a splicing event. Figure 1 illustrates the process of clustering short reads and determination of candidate splice site by MAW.

### Computational Methods

***Filtration of short reads.*** After MAWs were generated from genome sequences, a filter was applied on both short reads and MAWs. Short reads that are present or with their isoforms (with up to 2 mismatches from original short read) present in genome sequences (may correspond to novel exons) are filtered out. We emphasize that only short reads that are unique reads (or multi-reads which are not aligned to genome sequences or known gene models) would be retained after filtration. Filtration of multi-reads might lead to some mis-identification of splicing events that aligned to multi-reads, but since multi-reads have much higher false positive identification rates, we believe that this would not affect the sensitivity of splicing event identification.

**Figure 2:**
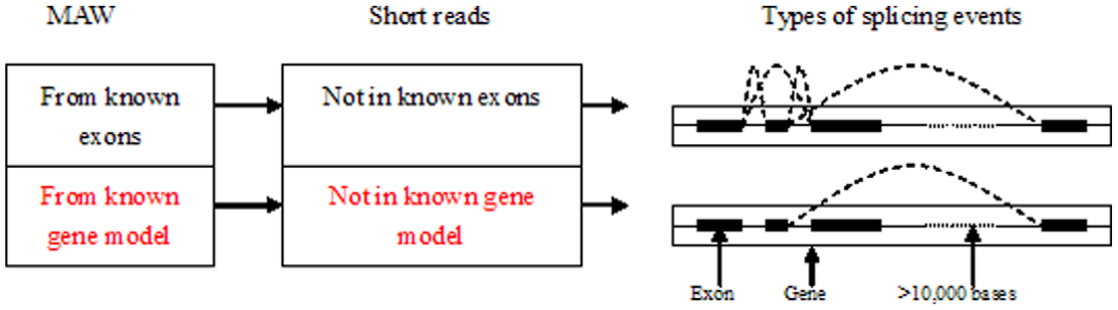
Different types of MAWs, the corresponding short reads and the splicing events that these short reads could identify. Note that these types have the inclusive relationship: MAWs from known exons include those from known gene model; the same for short reads and splicing event. Short reads that are not present in known gene models are likely to correspond to splicing events between distant exons.

***Clustering short reads according to MAWs.*** Different types of MAW correspond to different splicing events. Short reads that are not present in known exons might represent any kind of splicing events. Those that are also absent from known gene models might represent novel splicing events not in known gene model (Figure 2). In this work, we have focused on short reads for novel splicing events not present in known gene models.

Since each of the candidate splicing events correspond to a MAW (splicing event fingerprint), a set of short reads could be clustered together based on the corresponding MAW. Based on Lemma 2, it is easy to see that extracting MAWs only from the forward strand is enough to generate short read clusters. If a MAW is aligned (including reverse compliment) to a minimal number unique short reads (set to be 5 in this study), then the short reads for this MAW could form a short read cluster.

A consensus sequences was then generated from these short reads, which might correspond to splicing event. A consensus sequence of all short read in the cluster may not be accurate at the two ends. Therefore, we only take substring of consensus sequences in which each of the characters could be aligned to at least 5 short reads in the cluster (5-fold). Each of the short reads in the cluster should be aligned to the consensus sequence with at most 1 mismatch, otherwise it is removed from the cluster. Additionally, short read clusters could be merged if significant proportions of their consensus sequences are overlapping. Again, the merging criteria is that each of the characters in the consensus sequence after merging is aligned by least 5 short reads in the cluster (5-fold).

By mapping MAWs onto short reads and then clustering short reads, two objectives have been achieved before search of the genome is undertaken: (a) a relatively long consensus sequence for possible splicing junction has been obtained that is supported by multiple short reads; and (b) the candidate splicing sites on this consensus sequence. The most significant advantage of SAW is its ability to identify splicing events between distant exons without the cost of compiling junction library. This is not only the advantage in efficiency, but more importantly in reliable identification of splicing event without introducing an overwhelming amount of noise.

***Identification of splicing events.*** Consensus sequences from short read clusters could be used for the identification of all splicing events, including known splicing events and novel splicing events between distant exons. Given a consensus sequence and the corresponding candidate splicing sites, a candidate splice site would separate into prefix and suffix sequences. If the prefix of consensus sequence matches with the exon boundary of one exon, and the suffix of consensus sequence matches with the exon boundary of another exon, then this consensus sequence may correspond to a splicing event. For reliable splicing event identification, the prefix or suffix of consensus sequence that matches with exon boundaries (with up to 1 mismatch at either boundary) should be at least as long as 5 nucleic acids. This is illustrated in Figure 2.

***Statistical analysis of splicing events identification.*** Since SAW is able to identify thousands of splicing events, it is critical to statistically analyze the probability that the splicing event is a true positive rather than a random match of exon boundaries. To this end, negative exon models (decoy exon models) were constructed by cutting each of the exons in half, exchanging the prefix and suffix. The original exon is replaced with this modified sequence (Figure S1). A splicing event identified from negative exon models is a false identification. The overall false discovery rate (FDR) is computed as the ratio of the number of splicing events identifications from negative exon models, over the number of those from original exon models.

Moreover, to evaluate the accuracy of splicing event identification, the junction sequences for splicing events are aligned to EST sequences by BLAT, and E-values are then computed. Small E-values of these methods would indicate high quality hit to EST sequences. These E-values are also compared with those from searching randomly generated junction sequences.

**Figure 3:**
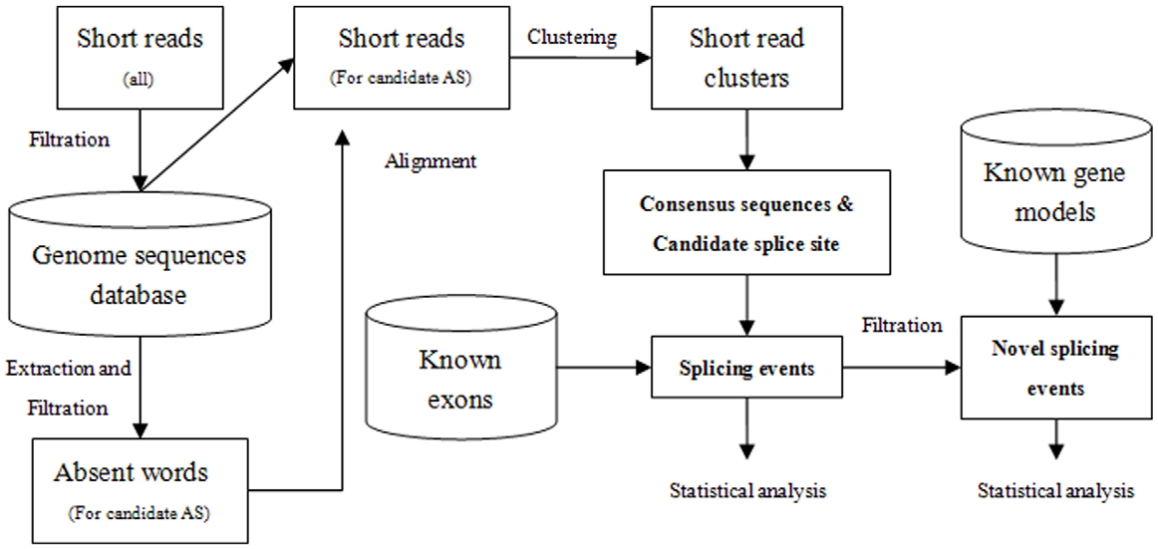
General scheme for splicing event identification by SAWs. Note that filtration against known gene models is only necessary for identification of novel splicing events.

The overall splicing event identification scheme is shown in Figure 3.

## Results

### Datasets

In this work, the mouse genome sequences from UCSC (mm9) have been used for splicing event identification. The MAWs were extracted from mm9 genome sequences. There were 873,257 MAWs generated from mm9 genome sequences. The distribution of these MAWs by length was illustrated in Figure S2. The short reads were obtained from recent RNA-Seq analysis of transcriptome of mouse brainstem tissues [11]. There were 47,781,892 short reads of length 25 (25-mers) for brainstem tissue. Decoy exon models were generated based on mouse mRNA sequences from mm9.

### Identification Results

***Alternative splicing on known gene models.*** First we have compared the splicing events identified by SAW with known splicing events in mm9 gene models. Here we have shown that based on short read clusters, SAW was able to filter out a large proportion of short reads while still retain high sensitivity.

Among 368,389 known splicing events, 226,583 were matched to at least 1 short reads, and 63,090 were matched by at least 5 short reads. After applying filtrations on short reads by comparing genome sequences, only 28,806,232 (60%) short reads left. After collecting the short reads to clusters of at least 5 short reads each, there were only 16,292,230 (34%) short reads left. More than 65% of the short reads were filtered out after short read clustering. For splicing event identification, when no mismatch was allowed between MAW and splice junction, based on short read clusters with at least 5 short reads, 70.6% (44,565/63,090) splicing events could be identified. However, there is no significant correlation of the MAW length and the proportion of the corresponding short read clusters that match to splicing junctions (Person correlation R<0.1).

**Figure 4:**
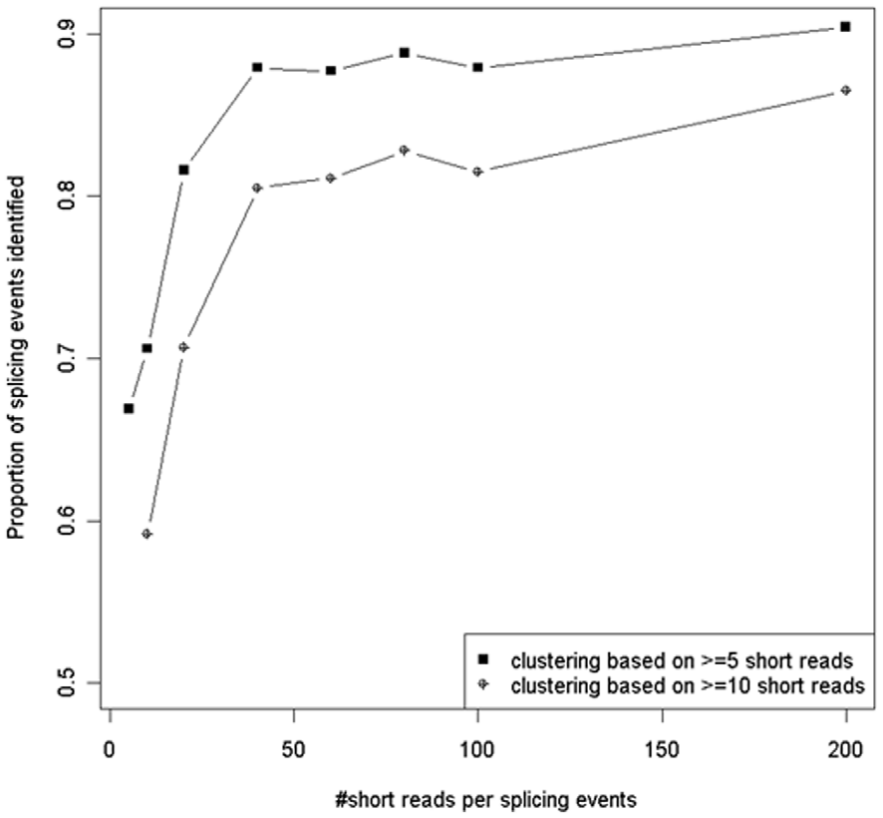
The increase of the proportion of splicing events identified by SAW with increasing number of short reads per splicing event. Results are based on short read clusters with minimum size of 5 and 10.

Additional analysis has shown that as the number of short reads per splicing event increased, the proportion of splicing events identified by SAW also increased (Figure 4). For splicing events with more than 200 short reads, over 90% could be identified by SAW. To further investigate the relation of minimum size of short read cluster and the proportion of known splicing events identified, we have set the smallest size of short read cluster to 10 (instead of the default 5). Based on this setting, only 14,403,116 short reads remain after clustering, and over 70% of the short reads were filtered out. In such a setting, the proportions of known splicing events identified by SAW were 10% less than those based on short read cluster of size ≥5 (Figure 4). Furthermore, no matter what setting of the minimum size of the short read cluster, the sensitivity of SAW increased significantly with the increase of allowed mismatches between consensus sequences and splicing junctions (Table 1). Among known splicing events that were not identified (323,824), around 45% were unidentified from short reads (allowing 1 mismatch), 50% did not have more than 5 short read alignments, around 3% corresponded to multi-reads, and the others 2∼3% were splicing events that were failed to be matched by consensus sequences.

**Table 1:** Sensitivities of short read clusters for identification of splicing events based on splicing events identified by ERANGE.

Mismatches allowed	Clusters by MAW with number of reads≥1	Clusters by MAW with number of reads≥5	Clusters by MAW with number of reads≥10
0	51.4%	21.7%	11.1%
1	100%	91.6%	55.2%
2	100%	99.0%	80.6%

**“Mismatches allowed” is referred to the maximum allowed mismatches between consensus sequences from short read clusters and splicing junction sequences.**

We have then compared MAWs with ERANGE [11] on the same datasets. If no mismatch was allowed, then 51.4% (105,532/205,151) of splicing events by ERANGE corresponded to at least one MAW. When two mismatches were allowed, all of 205,151 splicing events by ERANGE correspond to at least one MAW. Additionally, ERANGE have identified less than 60% of known splicing events, while almost all could be identified by MAWs with 2 mismatches (Table 1).

The false discovery rate (FDR) was also analyzed based on both forward (known) and decoy exon models, and it was discovered that 74 decoy splicing events would be identified by at least 5 short reads. However, base on short read clusters with at least 5 short reads, only 38 decoy splicing events would be identified. This corresponded to a FDR far below 1%.

***Predictions of novel splicing events.*** The power of SAW was not only on high sensitivity of identification of known splicing events, but more importantly the identification of novel splicing events between distant exons. We only focused on 7,000 splicing junctions identified by SAW between distant exons in the same genes and not present in known gene models. We considered these to be the candidate novel splicing events.

**Figure 5:**
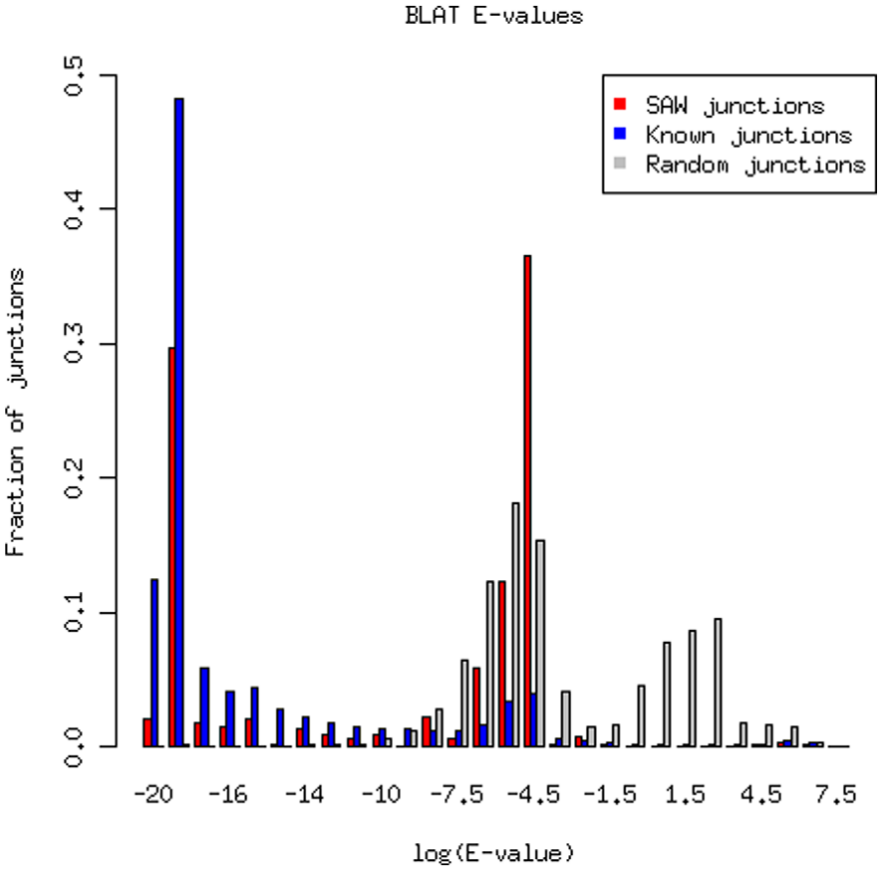
The BLAT E-value distribution of 48mers that spanning known junctions, random junctions and junctions identified by SAW.

To assess the quality of these predicted splicing events between distant exons, we have searched them against the mouse EST database from GenBank (downloaded as of 03/12/2010) using BLAT [14]. To compare the results, we have also searched the EST database for known junctions and decoy junctions. The known junctions included all junctions from mm9 gene models, and decoy junctions are composed of all junctions in decoy gene models. All of the sequences in these three groups were of length 48, which was the concatenation of 24 nucleic acids on from both of the exons involved in the junction. Figure 5 showed the distribution of E-values for these three groups. The majority of known junctions had high quality hits to EST sequences, while the majority of unknown junctions had low quality hits to EST sequences. For unknown junctions predicted by SAW, nearly 30% of these junctions had high quality hits to EST sequences (Figure 5).

**Figure 6:**
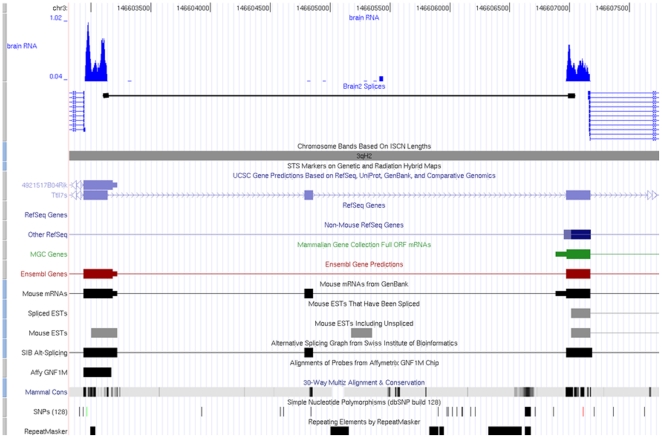
Example of novel splicing event identified by SAW from multiple reads in gene Ttll7s. The splicing events (annotated by black reads) are not identified by [11] based on UCSC mm9 gene models.

Several putative splicing events based on distant exons, whose presence was supported by extensive read coverage, were identified. Analysis of the gene expression profile based on RPKM [11] showed that for these novel splicing events, the corresponding exons also had high expression level. Examples of such splicing events were illustrated in Figure 6, Figure S3 and Figure S4.

### Efficiency of splicing event identification

All of the experiments were performed on a Linux server with eight 2.2 GHz Opteron cores and 16.0GB RAM. Generation of MAWs was based on linear time suffix tree search [13], and it took less than 10 minutes on mm9 mouse genome sequences. Filtration of MAW and of short reads was also very fast and were completed within an hour. The clustering and generation of consensus sequences by heuristic methods were also completed within an hour. Alignment of prefix and suffix of consensus sequences to exon boundaries with mismatches was the most time consuming part, but based on transforming exon sequences into Burrows-Wheeler indexing structure [10], [15], this was completed in less than 8 hours. Therefore, the whole process of identification of novel splicing events from short reads was within 10 hours on mm9 mouse genome sequences, which was quite efficient.

## Discussion

In this work, we have proposed a novel method, SAW, for efficient and accurate identification of novel splicing events. This method clusters short reads into splicing fingerprints (MAWs), generates a consensus sequence for each cluster, selects candidate splicing sites, and then aligns consensus sequences to gene models in search of splicing events. For alternative splicing events identified on known gene models, it was discovered that most of the known splicing events could be identified by SAW. We have also shown in our work that there are only a small proportion of splicing events correspond to multi-reads. There was no limitation on the vicinity of exons for the alignment of consensus sequences to exon boundaries by SAW. This implies that the method is especially useful for identification of novel splicing events between distant exons. In our experiments on mouse genome sequences, thousands of splicing events between distant exons, which were not in known gene models, were identified. Among these novel splicing events between distant exons, 30% of were of high quality.

We emphasis that SAW is designed for identification of splicing events, with special focus on splicing events between distant exons. Hence it is different from other splicing event identification methods. Another splicing event identification method, Tophat, is focused on the identification of all possible splicing events based on the known or predicted gene models. Since Tophat directly maps short reads to all putative splicing junctions for prediction, its results would have high sensitivity of splicing junctions between known or predicted exons. However, there is a limitation of the distance between two exons by this method based on all putative splicing junctions. By clustering short reads before mapping consensus sequences, the SAW method does not have such a limitation. On the dataset we have examined, 99% of known splicing events could be identified by MAWs with 2 mismatches (Table 1), indicating that SAW also has a very high sensitivity for splicing event identification. Furthermore, we have analyzed the effect of intron length to the number of splicing events identified by Tophat (data from [10]) and SAW. Results showed that while the number of splicing junctions by Tophat was decreasing with the increasing distance between exons, the results of SAW were not affected by the intron length. Additionally, from more than 10,000 short reads that were discarded by Tophat (unmappable to genome sequences or splicing junctions), many splicing junctions between distant exons were identified by SAW.

Because of its ability to identify novel splicing events, it is critical to generate accurate and error-tolerate consensus sequences for short read clusters, especially in cases where clusters might be merged. We have already shown that that allowing 1∼2 mismatch when comparing consensus sequences to junction sequences would leverage the genome errors fairly well on the dataset examined. However, sequencing errors, reference genome isoforms (e.g., SNP) and reference genome errors might introduce a large number of error when matching consensus sequences to splicing junctions. In this perspective, SAW could be further improved by a more sophisticated consensus sequence generation method, or by generating more than one consensus sequence per short read cluster. Another possible improvement of SAW might be the inclusion of multi-reads after short read clustering for additional splicing event identification.

In this study, the short reads from mouse brainstem tissue are used for identification of splicing events in mouse brainstem tissue. It is apparent that the splicing profiles are not the same in mouse brainstem and in other tissues such as mouse liver or skeletal muscle tissues. Investigating the different sets of short read clusters for these different tissues might provide additional information about the different splicing profiles in different tissues. The analysis of the differences in MAW sets generated from mouse genome sequences and other genome sequences such as for human might also provide interesting information about their different splicing profiles.

SAW is not only a standalone method for splicing event identification, but could also be embedded into other splicing event identification method as a short read filter to increase the speed and accuracy of splicing event identification. Since SAW is not limited by the distance between exons, another interesting application of SAW is probably the identification of fusion genes.

As fingerprints for splicing events, MAW could be combined with other next-generation sequencing techniques for efficient and accurate splicing event identification. For long reads (>500 bp) generated from other next-generation sequencing platforms (e.g. 454 sequencing technique), there is a problem of matching reads to multiple exons. In these situations, identification of splice site becomes a hard problem, which is extremely difficult for conventional splice junction library method. However, based on MAW, the candidate splice sites could be selected accurately without the information of gene models. Additionally, for pair-end reads alignment, MAW can be used to quickly check the splicing junctions before alignment, which could potentially improve the efficiency of splicing event identification.

Another avenue of research is the combination of genomic and proteomic data for the identification of novel splicing events. A splicing event may correspond to protein products if the corresponding transcripts are translated [16] and annotated in the database. On the other hand, there are many high quality mass spectra that do not correspond to any peptides in known protein sequences database. Therefore, searching high quality unassigned mass spectra against translated novel junction sequences would be beneficial on both sides: increasing the novel junction identifications, and increasing the number of spectra assignments. This approach based on combination of genomic and proteomic data would be further extended in applications such as fusion gene identification [17], [18].

## Supporting Information

Figure S1 The construction of decoy splicing events by exchange the prefix and suffix of the corresponding exons in real exon models (i). MAW matched to sequences in decoy exon models (ii) would equal to MAWs matched to a very rare, if at least possible, splicing event in real exon models (iii).
(0.40 MB TIF)

Figure S2 The number of minimal absent words from genome sequence of different length. Results were based on mm9 genome sequences.
(0.78 MB TIF)

Figure S3 UCSC snapshot of splicing events identified by SAW from multiple reads in gene Gpbp1L1. The splicing events (annotated by black reads) were not identified by ERANGE based on UCSC mm9 gene models. There are 12 short reads supporting this splicing event.
(0.92 MB TIF)

Figure S4 UCSC snapshot of splicing events identified by SAW from multiple reads in gene Mtap4. The splicing events (annotated by black reads) are not identified by ERANGE based on UCSC mm9 gene models. There are 16 short reads supporting this splicing event.
(0.84 MB TIF)
