# Supplementary figures and images for: SAW: A Method to Identify Splicing Events from RNA-Seq Data Based on Splicing Fingerprints
Source: PLoS One. 2010 Aug 10;5(8):e12047. doi: 10.1371/journal.pone.0012047 (PMC2919401; doi:10.1371/journal.pone.0012047)

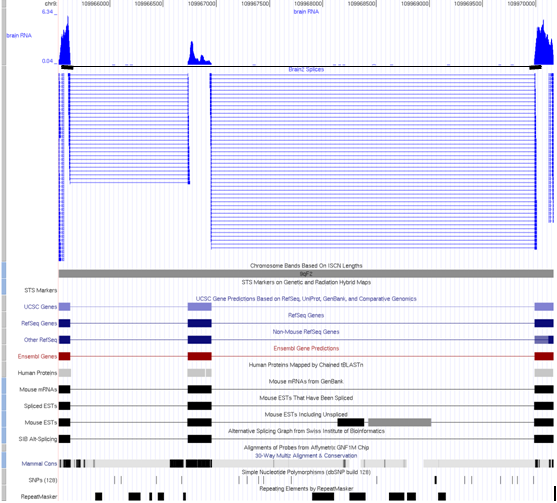

Supplement: Figure S1 — The construction of decoy splicing events by exchange the prefix and suffix of the corresponding exons in real exon models (i). MAW matched to sequences in decoy exon models (ii) would equal to MAWs matched to a very rare, if at least possible, splicing event in real exon models (iii). (0.40 MB TIF) [file pone.0012047.s001.tif]

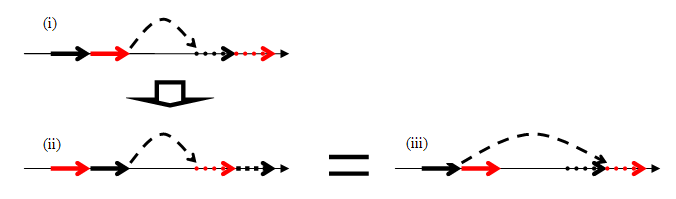

Supplement: Figure S2 — The number of minimal absent words from genome sequence of different length. Results were based on mm9 genome sequences. (0.78 MB TIF) [file pone.0012047.s002.tif]

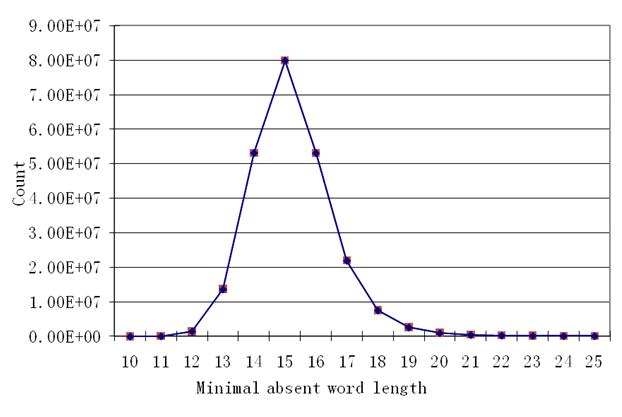

Supplement: Figure S3 — UCSC snapshot of splicing events identified by SAW from multiple reads in gene Gpbp1L1. The splicing events (annotated by black reads) were not identified by ERANGE based on UCSC mm9 gene models. There are 12 short reads supporting this splicing event. (0.92 MB TIF) [file pone.0012047.s003.tif]

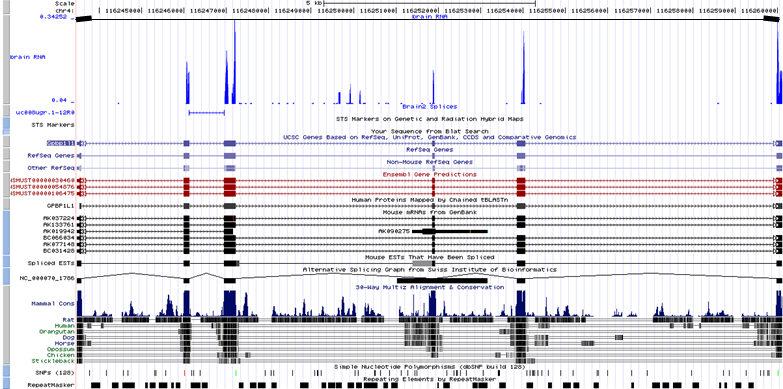

Supplement: Figure S4 — UCSC snapshot of splicing events identified by SAW from multiple reads in gene Mtap4. The splicing events (annotated by black reads) are not identified by ERANGE based on UCSC mm9 gene models. There are 16 short reads supporting this splicing event. (0.84 MB TIF) [file pone.0012047.s004.tif]
